# A receptor-antibody hybrid hampering MET-driven metastatic spread

**DOI:** 10.1186/s13046-020-01822-5

**Published:** 2021-01-14

**Authors:** Chiara Modica, Cristina Basilico, Cristina Chiriaco, Nicla Borrelli, Paolo M. Comoglio, Elisa Vigna

**Affiliations:** 1 Candiolo Cancer Institute, FPO-IRCCS, Strada Provinciale 142, 10060 Candiolo, TO Italy; 2 Department of Oncology, University of Turin, Turin, Italy

**Keywords:** MET, HGF, Metastasis, Targeted therapy, Fusion proteins

## Abstract

**Background:**

The receptor encoded by the MET oncogene and its ligand Hepatocyte Growth Factor (HGF) are at the core of the invasive-metastatic behavior. In a number of instances genetic alterations result in ligand-independent onset of malignancy (MET *addiction*). More frequently, ligand stimulation of wild-type MET contributes to progression toward metastasis (MET *expedience*). Thus, while MET inhibitors alone are effective in the first case, combination therapy with ligand inhibitors is required in the second condition.

**Methods:**

In this paper, we generated hybrid molecules gathering HGF and MET inhibitory properties. This has been achieved by ‘head-to-tail’ or ‘tail-to-head’ fusion of a single chain Fab derived from the DN30 MET antibody with a recombinant ‘ad-hoc’ engineered MET extracellular domain (decoyMET), encompassing the HGF binding site but lacking the DN30 epitope.

**Results:**

The hybrid molecules correctly bind MET and HGF, inhibit HGF-induced MET downstream signaling, and quench HGF-driven biological responses, such as growth, motility and invasion, in cancer cells of different origin. Two metastatic models were generated in mice knocked-in by the human HGF gene: (i) orthotopic transplantation of pancreatic cancer cells; (ii) subcutaneous injection of primary cells derived from a cancer of unknown primary. Treatment with hybrid molecules strongly affects time of onset, number, and size of metastatic lesions.

**Conclusion:**

These results provide a strategy to treat metastatic dissemination driven by the HGF/MET axis.

**Supplementary Information:**

The online version contains supplementary material available at 10.1186/s13046-020-01822-5.

## Background

The *MET* oncogene encodes for a tyrosine kinase, the high affinity receptor of hepatocyte growth factor (HGF). The HGF/MET axis drives a complex biological program known as ‘invasive growth’ including cell-cell dissociation (scattering), extracellular matrix degradation, dissemination at distant sites, cell proliferation, and survival [1]. These processes, pivotal during embryonal development, tissue homeostasis, and wound healing, are aberrantly harnessed by cancer cells during tumor progression [2, 3]. In a limited number of cases - 2-3%, COSMIC database [4] - the presence of a genetic lesion makes *MET* the ‘*driver’* gene of malignancy (MET *addiction*) [5, 6]. Nevertheless, activation of the MET kinase is relevant not only in cases of oncogene addiction: in fact, cancer cells exploit the MET-driven invasive growth program to arrange and sustain the adaptive response to adverse micro-environmental conditions. Through ligand activated MET signalling, tumors survive in hypoxia conditions [7], fight immune attacks [8, 9], resist radiotherapy [10], and sustain the clonal expansion occurring after resistance to targeted treatments [11, 12]. In these situations, MET, although not being a *driver*, represents an extremely useful ‘*expedient’* (MET *expedience*) for cancer cells survival and spreading [13]. MET *expedience* is pro-metastatic in a wide spectrum of tumor types, including colon-rectal, ovarian, and pancreatic cancers, and correlates with aggressive phenotype and severe clinical prognosis [14–16]. We previously demonstrated that concomitant targeting of HGF and MET has therapeutic efficacy in counteracting MET expedience [17]. Co-targeting was obtained by combining the activity of a soluble recombinant MET extracellular domain (decoyMET) with a MET antibody (MvDN30). DecoyMET is a recombinant protein featuring a dual inhibitory activity: on one side it binds HGF with high affinity; on the other, it dimerizes with full size MET receptors, working as a ‘*dominant negative*’ [18]. MvDN30 is an antibody endowed with a peculiar mechanism of action, the enhancement of the physiological rate of MET shedding [19]. Concomitant delivery of the two separate molecules – while somehow cumbersome - resulted in quenching of HGF and elimination of MET receptor from the cell surface, showing that simultaneous targeting of HGF and MET was non-redundant and synergic. In this study, we engineer a fusion protein assembling in a single agent the inhibitory properties toward the ligand and the receptor, providing a novel tool to fight metastatic spread driven by MET *expedience*.

## Methods

### Design and production of recombinant fusion proteins

Recombinant fusion proteins were designed starting from the sequence of MvDN30_long-sc60 [20] and decoyMET^K842E^ [17], joining the two moieties by an amino acid sequence (linker). DecAb fusion proteins were obtained connecting the IPT4 domain of decoyMET^K842E^ to the variable domain of the light chain of MvDN30_long-sc60. AbDec fusion proteins were generated linking the constant domain of the heavy chain of the antibody fragment to the α-chain of the SEMA domain of decoyMET^K842E^. At the C-terminus of each molecule, a triple Strep-tag (GAAWSHPQPEK) and a poly-histidine tag (HHHHHH) were added for protein purification and detection. cDNA synthesis, protein expression, and purification were performed by U-Protein Express BV (Utrecht, The Netherlands). Purified products were analyzed by SDS-PAGE followed by Gel Code Blue staining (Thermo Fisher Scientific, Waltham, MA, USA).

### Cell culture

A549 human lung adenocarcinoma cells, HPAF-II and Capan-1 human pancreatic adenocarcinoma cells were obtained from ATCC/LGC Standards S.r.l. (Sesto San Giovanni, Italy), and cultured as suggested by the supplier. Identity of the cell line was confirmed using DNA fingerprinting by short tandem repeat (STR) profiling (Promega PowerPlex® 16 System) and the Applied Biosystems Genotyper 2.0 software for analysis of the amplicons. M049 human metastatic colorectal cancer spheroids were kindly granted by Carla Boccaccio (University of Torino) and were maintained as described [21]. Primary cell line CL-901 was derived from the PDX-901 (Verginelli et al., 2020 submitted manuscript). The human sample was collected through the Agnostos program, a clinical and translational platform developed at the Candiolo Cancer Institute including a prospective, randomized phase-II clinical trial (NCT02607202), and the Agnostos Profiling study [22] approved by the Institute Ethical Committee. Informed consensus was obtained from patient, and the experiments were conformed to the principles set out in the WMA Declaration of Helsinki and the Department of Health and Human Services Belmont Report. Cancer of Unknown Primary diagnosis was made following the *ad-excludendum* diagnostic workflow, in accordance with ESMO guidelines [23]. After expansion in NOD-SCID immunodeficient mice, the tumor was excised, mechanically disaggregated, and digested with Collagenase I (Sigma-Aldrich, St. Louis, MO, USA). The recovered cells were cultured in Dulbecco’s Modified Eagle’s Medium/Nutrient Mixture F12 (DMEM/F12-Gibco) supplemented with 10% Fetal Bovine Serum (FBS, Sigma Aldrich). When confluent, monolayers were treated with trypsin: early-detaching cells were collected and kept in culture, while cells detaching later (mostly fibroblasts) were discarded; the procedure was repeated until the culture appeared clean from fibroblasts (14 passages). Authentication of the CL-901 cell line was performed by: (i) DNA fingerprinting by short tandem repeat (STR) profiling compared with peripheral blood mononuclear cells from the AGN_00901 Cancer of Unknown Primary patient; (ii) expression analysis of human vs mouse of Actinα1 and HPRT1genes; (iii) mutation analysis by checking the presence of LRP1B mutations previously detected in the patient by Sanger sequencing. All cell cultures were routinely tested for mycoplasma contamination.

### ELISA binding assays

For analysis of the interaction between the antibody domain of the fusion proteins and MET, wild type decoyMET (100 ng/well), produced by U-Protein Express BV, was immobilized on enzyme-linked immunosorbent assay (ELISA) plates and increasing concentrations (0–1 μM) of the fusion proteins were added in liquid phase. Binding was revealed using horseradish peroxidase (HRP)-conjugated anti-human k light chain antibodies (Sigma-Aldrich). For the analysis of the interaction between the decoy domain of the fusion proteins and HGF, DO24 anti-MET antibody [24] (100 ng/well) was immobilized on ELISA plates to capture the fusion proteins (5 nM) in solid phase; then, increasing concentrations (0–10 nM) of HGF (R&D Systems, Minneapolis, MN, USA) were added in liquid phase. Binding was detected using the anti-HGF biotinylated antibody BAF294 (R&D Systems) and revealed with HRP-conjugated streptavidin (GE Healthcare, Chicago, IL, USA). Colorimetric assay was quantified by the multi-label plate reader VICTOR-X4 (Perkin Elmer Instruments INC., Whaltman, MA, USA).

### Western blot analysis

For evaluation of the activity of hybrid proteins on HGF-dependent MET activation, A549 and CL-901 sub-confluent cell monolayers were treated with the fusion proteins (1 μM) during the 24 h of starvation, and then stimulated with HGF (100 ng/mL) for 15 min at 37 °C. Cell lysates were obtained using Laemmli buffer, and total protein concentrations were measured using Pierce BCA Protein Assay Kit (Thermo Fisher Scientific). Equal amounts of total proteins were resolved by SDS-PAGE, and transferred to 0.2 μm nitrocellulose Trans-Blot Turbo TM membranes (Thermo Fisher Scientific). For protein detection, the following antibodies were used: anti-MET phospho-Tyr1234/1235 (D26); anti-MET (D1C2); anti-HSP90 (C45G5) (all from Cell Signaling Technology). All antibodies were applied according to the protocols supplied by the manufacturers. After incubation with appropriate HRP-conjugated secondary antibodies (Jackson Immuno Research Europe Cambridge, UK) and the ECL reagent (Promega Corporation Madison, Wisconsin, United States), Western blot bands were detected by ChemiDoc Touch Imaging System (Bio-Rad, Hercules, CA, USA).

### Cell motility assay

HPAF-II cells (8000 cells/well) were seeded in 96-well plates in complete culture medium. After 6 h, two concentrations of the fusion proteins (0.2 and 2 μM) were added to the cells. After additional 24 h, cells were stimulated with HGF (6.25 ng/ml) for 20 h. Cells were fixed with 11% glutaraldehyde and stained with 0.1% crystal violet (Sigma-Aldrich). For real-time motility assay, HPAF-II cells (8000 cells/well) were seeded in E-plates (Roche Diagnostics, Mannheim, Germany) in complete culture medium. After 6 h, fusion proteins (3 μM) were added to the plate. 24 h later, cells were stimulated with HGF (6.25 ng/ml) for 18 h. Electrical impedance was monitored continuously for the entire duration of the experiment using the X-Celligence RTCA device (Roche Diagnostic, Mannheim, Germany). Data were recovered every 15 min and the values are expressed as Cell Index.

### Cell viability assay

Cell viability of M049 colon spheroids was performed by seeding the cells in 96-well plates (1000/ well) in stem cell medium in the presence of HGF (20 ng/ml). The following day, spheroids were treated with increasing concentrations of the fusion proteins (28,140 and 700 nM). After 4 days of treatment, cell number was determined using CellTiter-Glo (Promega Corp., Madison, WI, USA) with a VICTOR X4 multi-label plate reader.

### Invasion assay

HPAF-II (1.5 × 10^5^ /well) and CL-901 (10^5^/well) cells were suspended in serum-free culture in the upper compartment of transwell chambers (8.0 μm pore polycarbonate membrane insert-Corning Inc., NY, USA) pre-coated with 30 μg/well of Matrigel Reduced Growth Factors (Corning). Fusion proteins (1 μM) were also added in the upper compartment of the transwell. The lower compartment of the chamber for HPAF-II cells was filled with 2% FBS and HGF (12.5 ng/ml), while for CL-901 was added cell culture medium supplemented with 1% FBS and HGF (25 ng/ml). After 24 h, cells on the upper side of the transwell filters were mechanically removed, while cells migrated through the membrane were fixed with 11% glutaraldehyde and stained with 0.1% crystal violet. All images were captured with optical microscopes (Leica, Wetzlar, Germany). Images were quantified with Image-J software.

### Flow cytometry analysis

Cells were detached with PBS/EDTA 1 M and stained with APC-conjugated mouse anti-Human MET (3D6; BD Biosciences, Allschwil, Switzerland). For isotype control, an APC-conjugated anti-mouse Ig antibody (BD Biosciences) was used. Cells were co-stained with DAPI. MET expression was analysed by Summit 4.3 software (Dako, Santa Clara, CA, USA). The signal derived from the isotype control was set as: 0 < MFI < 10^1^,and cells were considered MET positive when MFI > 10^1^.

### In vivo experiments

All in vivo experiments were performed according to protocols approved by the Ethical Committee for animal experimentation of the Fondazione Piemontese per la Ricerca sul Cancro and by the Italian Ministry of Health. hHGF-ki SCID mice were obtained from AVEO Pharmaceuticals, (Cambridge, MA, USA). For expression of the luciferase gene, cancer cells were transduced with 100 ng/ml p24 of lentiviral vectors carrying the luciferase gene under the control of the CMV promoter as described [25, 26]. For the pancreas orthotopic experimental model, luciferase-expressing HPAF-II or Capan-1 cells (10^5^ cells/mouse in 50 μl of phosphate-buffered saline) were injected in the pancreas of 4 to 6 week old female hHGF-ki SCID mice. After 3 days, XenoLight D-Luciferin (150 mg/kg; Perkin Elmer) was injected intraperitoneally in mice. Based on the bioluminescence signal measured by an IVIS Spectrum CT apparatus (IVIS, Perkin Elmer), mice were stratified into homogeneous groups and randomly assigned to the treatment arms. Fusion proteins (10 mg/kg) were administered twice weekly by intraperitoneal injection. The combination was administered as follows: MvDN30, 10 mg/kg daily; decoyMET^K842E^, 10 mg/kg every two days. Both proteins were produced and purified by U-Protein Express BV. At sacrifice, after five weeks of treatment, XenoLight D-Luciferin was administered to mice and the bioluminescent signals of tumors and isolated organs were measured by IVIS. For Cancer of Unknown Primary metastatic model, Luciferase-expressing CL-901 cells (2× 10^6^ cell/mouse in 200 μl of PBS supplemented with 20% Matrigel Matrix - Corning) were injected subcutaneously in the right flank of female hHGF-ki SCID mice. After three days, mice were divided in two groups and randomly assigned to the treatment arms: vehicle (PBS) or AbDec-L1 (50 mg/kg) twice a week by intraperitoneal injection. After five weeks of treatment, mice were injected with XenoLight D-Luciferin, euthanized, and isolated organs were analysed as described above. All IVIS data were analysed by Living Image software (Perkin Elmer). Immunohistochemical analysis was performed on primary tumors. Briefly, tumors were excised, fixed and paraffin-included. Slides were stained with AF2480 anti-human/mouse phosphoMET^Tyr1234–1235^ polyclonal antibodies (R&D System). Quantification of the staining was done by ImageJ software.

### Pharmacokinetic analysis

The study has been performed by Accelera S.r.l. (Nerviano, Italy). All the animal procedures were performed according to the current Italian legislation (Legislative Decree March 4th, 2014 n. 26) enforcing the 2010/63/EU Directive on the protection of animals used for biomedical research. AbDec-L1 has been administered by single intravenous injection to adult female Sprague Dawley rats (*n*=3) at the dose of 20 mg/kg. The serum levels of the molecule were evaluated at pre-dose and after 5 min, 30 min, 1, 4, 8, 24, 48, 72, 168 and 240 h post-dosing. The compound in serum was analyzed by an ELISA assay based on solid phase extraction with DO24 anti-MET antibody (2.5 μg/mL). Calibration curve was obtained loading different concentrations of the purified AbDec-L1 (range 0.0425–47.6 μg/mL) diluted in rat serum. AbDec-L1 was revealed using horseradish peroxidase (HRP)-conjugated anti-human k light chain antibodies. The pharmacokinetic profile of the compound was analyzed by standard non compartmental analysis using Phoenix-WinNonlin package (v. 8.1, Certara Company, USA). Samples at time 0, 48, 72, 168, and 240 h post-dosing were below the lower limit of quantification.

### Statistical analysis

Average and Standard Deviation (SD) were calculated using Microsoft Office Excel 2010 software (Microsoft Corporation, Redmond, WA) or GraphPad Prism Software. To calculate Kd values, data from ELISA assays were analyzed and fitted according to nonlinear regression, one site binding hyperbola curve, using GraphPad Prism software. Statistical significance was determined using the two-tailed Student’s T test or Mann-Whitney test. A value of *p* ≤ 0.05 was considered significant.

## Results

### Design of receptor-antibody hybrid proteins

To generate a recombinant fusion that is efficiently produced and assembled, the antibody must be in single chain format. We previously designed and validated MvDN30_long-sc60, a single chain Fab derived from DN30 that maintains MET inhibitory properties of the precursor antibody [20]. To prevent the reciprocal neutralization of the moieties by intra-chain binding, the MET epitope bound by DN30, the lysine residue at position 842 [17], was converted into a glutamic acid (decoyMET^K842E^). The recombinant molecules were then assembled by fusing the single chain Fab to decoyMET^K842E^ via an amino acid linker. As the best functional architecture of a fusion protein is hardly predictable, a panel of hybrids was generated, and their activity tested in different assays. We used six linkers differing in length, amino acid composition, and secondary structure (Table 1): (i) three flexible linkers (L1-L2-L3); (ii) two linkers with a rigid backbone (L4-L5); and (iii) one long sequence combining flexible and rigid structures (L6).

**Fig. 1:**
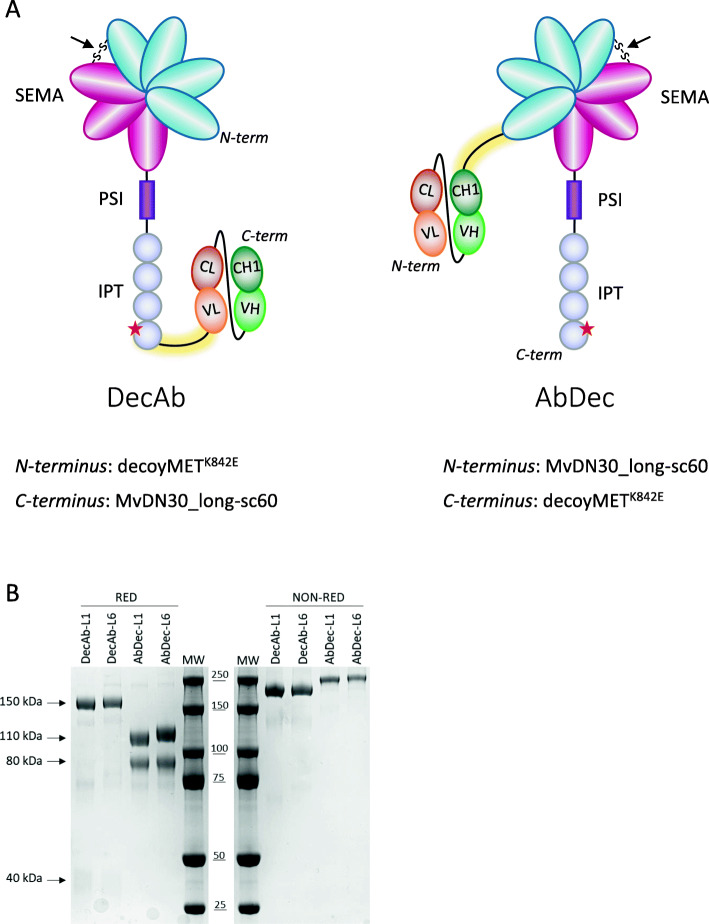
Design and generation of receptor-antibody hybrid proteins. **A** - Schematic representation of the receptor-antibody hybrid proteins featuring the decoyMET moiety at the N-terminus and the single chain MET antibody at the C-terminus (DecAb, left panel), or the single chain antibody moiety at the N-terminus and the decoyMET at the C-terminus (AbDec, right panel). SEMA, SEMA domain of MET (light blue: blades 1–4, corresponding to MET α-chain; magenta: blades 5–7). The black arrow indicates the disulfide bond linking α- and β-chains of MET; PSI, Plexin/Semaphorin/Integrin domain of MET. IPT, Immunoglobulin-like/Plexin/Transcription Factor domain of MET. The red star indicates a single point mutation (K842E) inserted in decoyMET to abrogate MvDN30 binding. VL-CL, Variable Light and Constant Light chains of MvDN30; VH-CH1, Variable Heavy and first Constant Heavy chains of MvDN30. The linker between the two moieties of the fusion molecule is highlighted in yellow. B - Coomassie Blue staining of affinity-purified hybrid proteins resolved by SDS-PAGE under reducing (RED) or non-reducing (NON-RED) conditions. In reducing conditions, DecAb hybrid proteins generate two bands of about 40 kDa (MET α-chain) and 150 kDa (MET β-chain + linker + single chain antibody), while AbDec hybrid proteins generate two bands of about 110 kDa (single chain antibody + linker + MET α-chain) and 80 kDa (MET β-chain)

For each linker, two recombinant fusion proteins swapped ‘head to tail’ were constructed: decoyMET^K842E^-linker-MvDN30_long-sc60 (named DecAb) or MvDN30_long-sc60-linker-decoyMET^K842E^ (named AbDec) (Fig. 1a). All molecules were screened by yield of production, purity, and correct assembly. By these criteria, four leads were selected: AbDec and DecAb-L1; AbDec and DecAb-L6. The hybrids were analyzed by SDS-PAGE in reducing and non-reducing conditions to verify the purity and the correct size (Fig. 1b).

**Table 1:** Linkers connecting the two moieties in the hybrid proteins

Name	Length	Amino acid composition	Features
**L1**	60	GGSSGSGSGSTGTSSSGTGTSAGTTGTSASTSGSGSGGGGGSGGGGSAGGTATAGASSGS	flexible
**L2**	17	GGGGSGGGGSGGGGSGG	flexible
**L3**	32	GGGGSGGGGSGGGGSGGGGSGGGGSGGGGSGG	flexible
**L4**	22	AEAAAKEAAAKEAAAKEAAAKA	rigid
**L5**	45	AEAAAKEAAAKEAAAKEAAAKAAAEAAAKEAAAKEAAAKEAAAKA	rigid
**L6**	134	GGGGSGGGGSGGGGSAEAWYNLGNAYYKQGDYQKAIEYYQKALELDPNNAEAWYNLGNAYYKQGDYQKAIEYYQKALELDPNNAEAWYNLGNAYYKQGDYQKAIEDYQKALELDPNNGGGGSGGGGSGGGGSGG	mixed (flexible/rigid)

### Functional validation in vitro

**Fig. 2:**
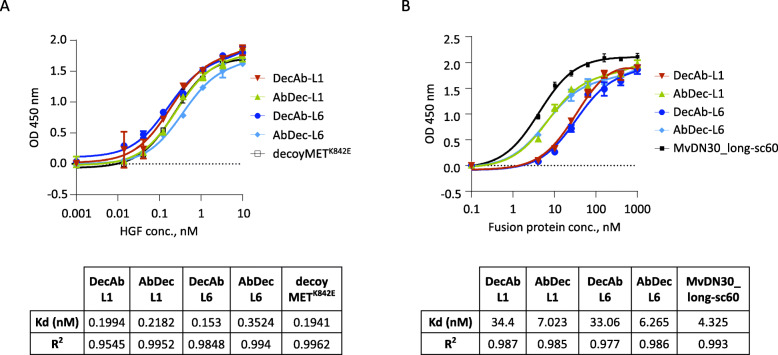
Binding analysis of the receptor-antibody hybrid proteins. **A -** Binding of the hybrids to HGF. DO24 anti-MET (100 ng/well) was used as capture antibody for purified recombinant fusion proteins (5 nM) in solid phase; increasing concentrations of HGF (0–10 nM) were added in liquid phase. The decoyMET^K842E^ was used as control. **B** - Binding of the hybrids to decoyMET. Purified decoyMET (100 ng/well) was in solid phase, and increasing concentrations of the recombinant fusion proteins (0–1000 nM) were added in liquid phase. The MvDN30_long-sc60 was used as control. Kd values are reported below each graph. Each point is the mean of values in triplicate ± SD. Data reported in the figure are representative of one experiment out of two

The selected hybrids were tested for their ability to interact with the ligand and the receptor, through the decoy or the antibody moiety, respectively. All hybrids bound HGF with high affinity, comparable to that displayed by the parental decoyMET^K842E^ itself (Fig. 2a). On the other hand, the hybrids displayed different binding properties towards MET. The molecules harboring the antibody moiety at the C-terminal (DecAb) displayed a reduced affinity, significantly lower than both MvDN30 and the molecules harboring the antibody moiety at the N-terminal (AbDec) (Fig. 2b).

**Fig. 3:**
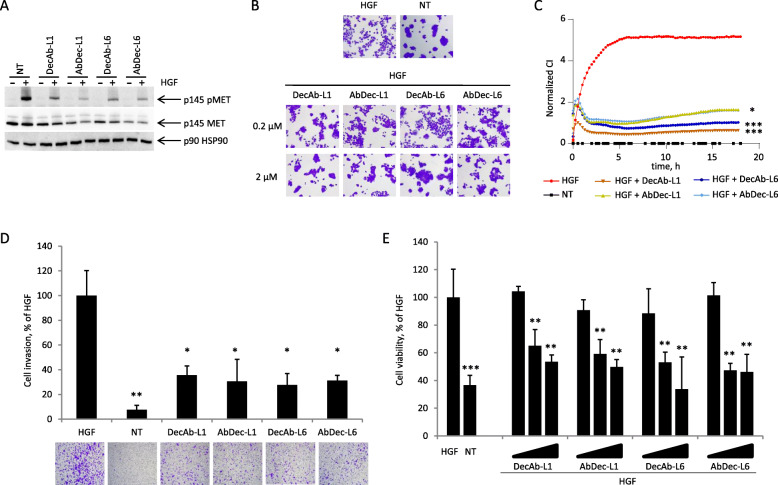
Receptor-antibody hybrid proteins inhibit HGF-dependent MET activation and biological responses. **A** - Hybrid proteins inhibit HGF-induced MET phosphorylation. A549 cells were incubated with 1 μM of each fusion protein for 24 h and then stimulated with 100 ng/ml of HGF for 15 min. p145 pMET, phosphorylated MET β-chain; p145 MET, MET β-chain; p90, HSP90. **B** - Hybrid proteins inhibit HGF-induced cell scattering. Representative images of HPAF-II cells treated with 0.2 or 2 μM of each fusion protein for 24 h and then stimulated with 6.25 ng/ml of HGF. NT, not treated cells. Magnification, 40x **C -** Cell scattering monitored in real time using an X-CELLigence RTCA device and expressed as normalized Cell Index (CI). HPAF-II pancreatic cancer cells were plated in E-plate wells, and then treated with 6.25 ng/ml HGF alone or in presence of 3 μM of the hybrid proteins. **D** - Hybrid proteins inhibit HGF-induced cell invasion. HPAF-II cells were stimulated with 12.5 ng/ml of HGF and treated with 1 μM of each hybrid protein. Graph represents the percentage of invasion in comparison with the HGF-stimulated cells. Each point is the mean of values in duplicate ± SD. One representative image/group of the cells migrated through the matrigel layer is showed below the graph. NT, not treated cells. Magnification, 25x. **E** - Hybrid proteins impair the viability of patient-derived colon cancer spheroids. M049 colon spheroids were pre-incubated with 20 ng/ml HGF and treated with increasing concentrations (0–700 nM) of each hybrid. Cell viability was determined by measuring cellular ATP. Graph represents the percentage of viability in comparison with the HGF-stimulated control. Each point is the mean of values in triplicate ± SD. ***, *p*≤ 0.001; **, *p*≤ 0.01; *, *p*≤ 0.05, ns: not significant. Data reported in the figure are representative of one experiment out of three

Then, the ability of the hybrids to inhibit HGF-dependent MET activation was evaluated. In the presence of the new recombinant proteins, the level of receptor phosphorylation was impaired with comparable potency (Fig. 3a).

The properties of DecAbs and AbDecs were further investigated in different biological assays, namely the inhibition of HGF-induced scattering and invasion of HPAF-II cells, a model of spontaneous metastatic pancreatic human carcinoma. These cells grow in compact islands, but in the presence of HGF they move away from each other, change morphology and spread. The receptor-antibody hybrids dose-dependently reverted cell scattering with comparable efficacy (Fig. 3b). To better assess the potency of the inhibitors, cell scattering was quantified by measuring the variations in electrical impedance of cell-covered electrodes (X-CELLigence Real Time Cell Analyzer). Cell index values measured at the end of the experiment (18 h after HGF addition) indicated that the four molecules reduced the HGF effect by 70–88% (Fig. 3c). Similar results were obtained in invasion assays performed on Matrigel-coated chambers: the hybrids impaired HGF-driven invasion of HPAF-II cells by 64–72% (Fig. 3d). Finally, we studied cell viability of M049 colon spheroids prepared from a patient-derived xenograft of colorectal cancer liver metastasis [21]. In this model, the hybrids counteracted in a dose-dependent manner the boost of cell growth induced by exogenously added HGF (Fig. 3e).

In conclusion, all hybrids displayed effective inhibitory activity in the biological assays tested, showing that the four selected molecules were functional.

### Therapeutic activity in preclinical models

The activity of the hybrid proteins was then tested in vivo in two models of spontaneous metastasis. The first model was built by orthotopic transplantation of human pancreatic ductal adenocarcinoma cells, and the second by subcutaneous transplantation of a primary cell line derived from a patient affected by a cancer of unknown primary. As it is known that the human MET receptor is not fully activated by murine HGF [27–29], the host SCID mice where knocked-in by the human HGF gene (hHGF-ki) [30]. This system fullfills the requirement for MET activation in conditions of expedience, and allows to evaluate the contribution of the HGF/MET axis to the dissemination of human cancer cells.

**Fig. 4:**
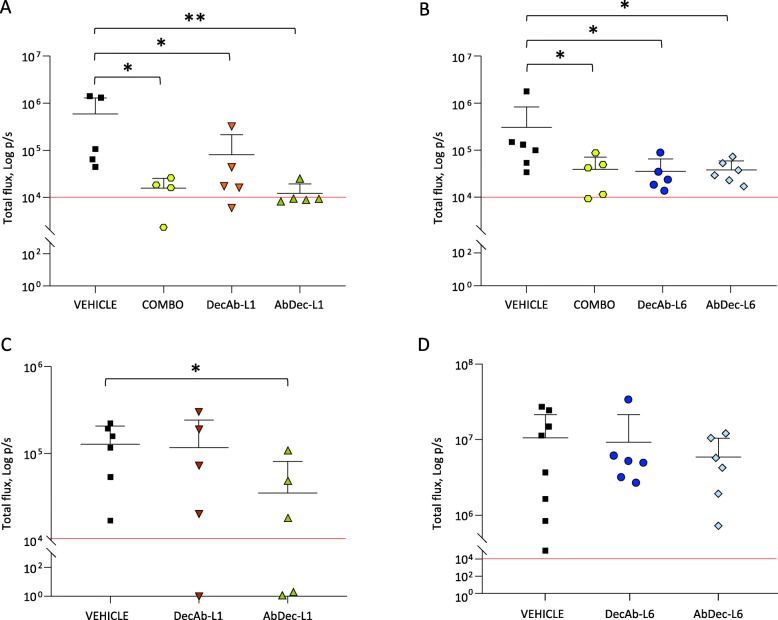
Receptor-antibody hybrid proteins inhibit pancreatic cancer cell dissemination in vivo. IVIS analysis of livers (**A-B**) or lungs (**C-D**) excised from hHGF-ki mice that received intra-pancreatic injections of Capan-1 or HPAF-II pancreatic cancer cells, respectively. Mice were treated with VEHICLE, the indicated hybrid proteins, or the combination of MvDN30 and decoyMET^K842E^ (COMBO). Each dot represents the value of the organ excised from one mouse. The red line indicates the threshold (10^4^) below which IVIS values are considered negative. **, *p* ≤ 0.01; *, *p* ≤ 0.05. Data reported in the figure are representative of one experiment out of two

Two different pancreatic cancer cell lines, Capan-1 and HPAF-II, genetically modified by lentiviral vectors to express luciferase, were injected in the pancreas of hHGF-ki mice. At sacrifice, primary tumor size and the presence of metastatic nodules in excised organs were checked by an In Vivo Imaging System (IVIS). The sites of preferential dissemination of each cancer cell line were analyzed, namely the liver for Capan-1 [31] and the lungs for HPAF-II cells [17]. The experiments were designed to directly compare DecAb and AbDec with the same linker. In both pancreatic cancer systems, the treatments had no substantial effect on primary tumor growth (Supplementary Fig. 1). On the contrary, metastatic dissemination was deeply affected by the hybrid molecules. In the Capan-1 experimental model, all hybrids significantly impaired liver colonization, similarly to what observed after treatment with the combination of MvDN30 and decoyMET^K842E^, the two parental molecules (Fig. 4a and b, Supplementary Fig. 2). AbDec-L1 scored as the best inhibitor: only one mouse out of five presented nodules in the livers, and its IVIS signal was reduced by 16.4 folds compared to the average values of the untreated group. In the HPAF-II model, only AbDec-L1 significantly counteracted cancer cell dissemination to the lungs (Fig. 4 c and d, Supplementary Fig. 3). Two out of five mice did not show any IVIS-detectable signal, and the values measured in the other three animals were lower than the average value of the untreated group. Inhibition of MET activation by AbDec-L1 treatment was confirmed in primary tumors (Supplementary Fig. 4).

**Fig. 5:**
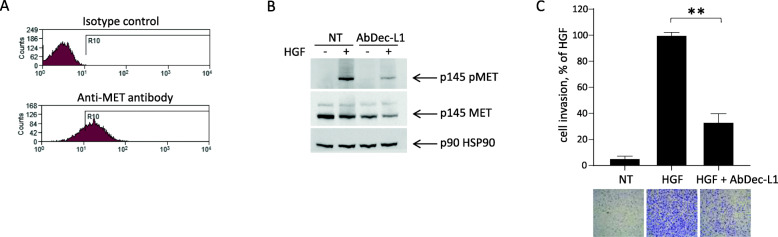
AbDec-L1 inhibits MET activation and invasion of CL-901 in vitro. **A** - Cytofluorimeter analysis of CL-901 cells stained with anti-MET antibodies. **B** -Immunoblotting analysis of HGF-induced MET phosphorylation in CL-901 cells treated with 1 μM AbDec-L1 and stimulated with 100 ng/ml HGF. Total cell lysates were immunoblotted with anti-phosphoMET (upper panel), anti-MET (middle panel) or anti-HSP90 antibodies (lower panel). **C** - Transwell invasion assay. CL-901 cells were stimulated with 25 ng/ml HGF and then treated with 1 μM AbDec-L1. Bars represent the percentage of invasion in comparison to the HGF-stimulated control. The figure panel below the graph shows one representative image/group of the cells migrated through the matrigel layer. Magnification, 25x. Each point is the mean of values in triplicate. Data reported in the figure are representative of one experiment out of three

The second model exploited the property of a primary cell line (CL-901, derived from a Cancer of Unknown Primary patient) to spontaneously disseminate to multiple mouse organs. The purity of the primary cell culture, established from a mouse xenograft, was assessed by real time PCR, using probes specific for human or mouse genes. Moreover, the coherence with the patient genetic traits was confirmed by finger print analysis, and by the presence of point mutations characterizing the original cancer lesion (data not shown). In vitro analysis of CL-901 cells showed that MET is expressed and correctly exposed on the cell surface (Fig. 5a). Administration of AbDec-L1 strongly impaired HGF-induced MET phosphorylation (Fig. 5b) and powerfully inhibited HGF-induced cell invasion (Fig. 5c).

**Fig. 6:**
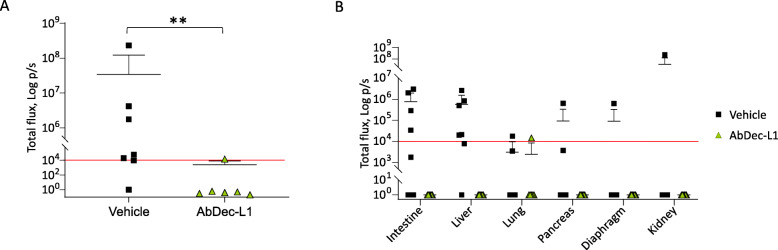
AbDec-L1 inhibits CL-901 dissemination in vivo. IVIS analysis of organs excised from hHGF-ki mice that received subcutaneous injection of CL-901 cells. Animals were treated with vehicle or AbDec-L1; organs were collected 31 days after cell injection. In **A**, each point represents the total IVIS signal detected for each mouse. In **B**, the organ distribution of the IVIS signal for each mouse is reported. The red line indicates the threshold (10^4^) below which IVIS values are considered negative. **, *p* ≤ 0.01. Data reported in the figure are representative of one experiment out of two

CL-901 cells were genetically modified by lentiviral vector to express luciferase and then injected subcutaneously in hHGF-ki mice. After three days, the animals were randomly assigned to two experimental arms: one group of mice received the vehicle, while the other was treated with AbDec-L1 by intraperitoneal injection. After 31 days, mice were euthanized and main organs were excised and analysed by IVIS. CL-901 cells displayed a highly metastatic behavior: 6 out of 7 mice of the control group showed detectable metastatic lesions; the most colonized organs were liver and intestine, but also pancreas, kidneys, diaphragm, and lungs were affected. On the contrary, mice treated with AbDec-L1 did not show signs of cancer cell dissemination, except for a very small nodule in the lung of one mouse (Fig. 6a and b, Supplementary Fig. 5). As observed in pancreatic tumors, also in this experimental model treatment with the hybrid impaired MET activation in the primary tumors (Supplementary Fig. 6a), while it did not influence their size (Supplementary Fig. 6b).

Finally, we analyzed the pharmacokinetic prolife of AbDec-L1 by delivering the hybrid as a single intravenous bolus at the dose of 20 mg/kg to female rats. Serum concentration of the molecule declined according to a poly-exponential curve (Supplementary Fig. 7). The pharmacokinetic profile was characterized by a terminal half-life of 4.35 ± 1.94 h, a serum clearance of 1.72 ± 0.17 ml/h/kg, and a volume of distribution at steady state and at terminal phase of 87.9 ± 26.3 and 645 ± 292 ml/kg, respectively.

## Discussion

The HGF/MET ligand/receptor pair, unleashing invasive and pro-survival cues, is a good candidate for a precision medicine approach to metastasis treatment. Here we describe design and validation of a new therapeutic tool based on genetic fusion of the HGF-neutralizing factor decoyMET^K842E^ with the MET antagonist antibody MvDN30, in single chain format. The new protein fully incorporates the functions of each component and has intrinsic advantages: (i) fixed stoichiometry between the two moieties; (ii) dosage and schedule optimization; (iii) effortless manufacturing; (iv) painless procedures for registration. In this respect, administration of one single molecule versus the two separated components ensures binding of both at the right rate on target cells. The antibody portion included in the hybrid is a monovalent single chain Fab. A variety of antibody-derived fragments, ranging from single domain antibodies to Fabs, have been used in therapy. They maintain the high specificity of the progenitors, conjugated with: (i) increased ability to penetrate tissues; (ii) monovalent binding; (iii) low-cost production in bacterial systems [32–34]. Nevertheless, antibody fragments have drawbacks, among which fast kidney clearance as consequence of their small size [35]. In the receptor-antibody hybrid described here, MvDN30 in single chain format is conjugated with decoyMET, and the resultant molecule has a size well above the glomerular filtration cut-off. Indeed, the schedule of administration applied to the hybrid is less frequent compared to the daily administration required for MvDN30 in combination with decoyMET^K842E^ [17]. According to the pharmacokinetic parameters, the hybrid is characterized by a low clearance, a short terminal half-life and a low volume of distribution at steady state that increases during the terminal phase. This indicates that the molecule is distributed to a peripheral compartment, where it remains available for blocking HGF/MET signaling, as supported by the outcome of the in vivo tests.

The second component of the hybrid here described is a ‘decoy*’* receptor. This class of recombinant molecules, lacking the signal transduction machinery (e.g. the tyrosine kinase domain), binds and sequesters cognate ligands, preventing activation of the naive cellular receptors. In most cases, decoy receptors have been fused to an antibody-derived Fc domain of irrelevant specificity [36, 37] simply to improve stability of the compound and to simplify production and purification processes. On the contrary, in the receptor antibody hybrid here described both components exert an intrinsic inhibitory activity, and work together to achieve dual targeting of the ligand and receptor pair.

Last but not least, the receptor-antibody hybrid overcomes the need of a double effort in production and purification of each single component, and simplifies the procedures to get approval for clinical applications.

Currently, the main strategies pursued to concomitantly inhibit two different targets is the application of bispecific antibodies [38–40]. With regard to MET receptor, a number of bispecific antibodies are under investigation. Most of them have been designed to simultaneously target multiple signaling pathways (e.g. MET/EGFR, MET/PD1, MET/EpCam) [41–44]. Two MET biparatopic antibodies feature two binding domains interacting with distinct epitopes of the same molecule. The first antibody targets an epitope located between PSI and IPT1 domains, and a second located in the SEMA domain [45]. The inhibitory property of this molecule relays on competition with HGF, and thus does not interfere with ligand-independent receptor activation (such as overexpression). The second MET-biparatopic antibody recognizes two separate epitopes on the SEMA domain [46] and acts by interfering with MET recycling at the cell surface.

Looking for an optimal design of the hybrid molecule, we tested different amino-acid linkers connecting the decoy and the antibody moieties, and tried opposite arrangements of the proteins, swapping the molecules located at the N- and C-terminus. All hybrids displayed superimposable HGF binding affinity, while AbDec molecules bound MET with higher affinity compared to DecAb molecules. This suggests that the spatial position and orientation of MvDN30 and decoyMET^K842E^ influence the interaction between the antibody moiety and MET. Likely, the antibody at the N-terminus provides optimal presentation of the epitope binding site, allowing freedom of spatial movements to correctly interact with MET. On the contrary, HGF binding sites of the decoy moiety remain available in all arrangements tested. Nevertheless, these differences in the binding affinity do not affect the in vitro biological activity of the hybrids. This indicates that, beyond ligand neutralization, the decoy component may exert further activities, enhanced in the N-terminal conformation, counterbalancing the lower antibody binding. In particular, the decoy could impair MET mediated biological responses by forming inactive heterodimers with full-length transmembrane MET receptors, and/or with other surface molecules involved in the amplification of MET signalling [47–49].

In vitro analysis did not allow the identification of a receptor-antibody hybrid conformation associated with a better performance. Otherwise, in vivo testing uncovered differences in preventing spreading of pancreatic cancer cells from orthotopic primary tumors. The mechanistic explanation of this therapeutic efficacy is unknown, however a good performance in vivo of AbDec-L1 was also observed in the second model generated by primary cancer cells derived from a Cancer of Unknown Primary patient. As expected by the highly aggressive nature of this pathology [50], these cells are intrinsically endowed with a strong dissemination capacity, testified by their uncommon property to form distant metastasis from a subcutaneous implant. The results obtained were particularly significant as both models were refined by using hHGF-ki immunocompromised mice, to fully estimate the contribution of the HGF-MET axis to the metastatization process.

## Conclusions

The new receptor-hybrid molecule here described effectively restrains metastasis dissemination driven by the HGF/MET axis, optimally blocking the pathway. The encouraging results obtained in two different preclinical models of spontaneous metastatic tumors provide proof of concept for potential clinical application of this experimental anti-metastatic tool.

## Supplementary Information

**Additional file 1: Supplementary Fig. 1.** IVIS analysis of primary tumors excised from mice that received intra-pancreatic injections of Capan-1 or HPAF-II pancreatic cancer cells.

**Additional file 2: Supplementary Fig. 2.** IVIS images of livers excised from hHGF-ki mice that received intra-pancreatic injection of Capan-1 cells.

**Additional file 3: Supplementary Fig. 3.** IVIS images of lungs excised from hHGF-ki mice that received intra-pancreatic injection of HPAF-II cells.

**Additional file 4: Supplementary Fig. 4.** Immunohistochemical analysis of MET phosphorylation in pancreatic primary tumors treated with AbDec-L1.

**Additional file 5: Supplementary Fig. 5.** IVIS images of organs excised from hHGF-ki mice that received sub-cuteaneous injection of CL-901 cells.

**Additional file 6: Supplementary Fig. 6.** Analysis of CL-901 primary tumors treated with AbDec-L1.

**Additional file 7: Supplementary Fig. 7.** Serum concentration of AbDec-L1 after single i.v. administration to Sprague Dawley rats at different time points.

## Data Availability

All data related to this study are included in this paper and its supplementary information files. No data sets were generated or analysed during the current study.

## Abbrevations

EGFR: Epithelial Growth Factor Receptor
hHGF-ki: SCID mice knocked in by the human HGF gene
HGF: Hepatocyte Growth Factor
IVIS: In Vivo Imaging System
IPT: Immunoglobulin-like/Plexin/Transcription Factor domain
PSI: Plexin/Semaphorin/Integrin domain
