# Supplementary material for: A receptor-antibody hybrid hampering MET-driven metastatic spread
Source: J Exp Clin Cancer Res. 2021 Jan 14;40:32. doi: 10.1186/s13046-020-01822-5 (PMC7807714; doi:10.1186/s13046-020-01822-5)
Supplement: Supplementary file 1 — Additional file 1: Supplementary Fig. 1. IVIS analysis of primary tumors excised from mice that received intra-pancreatic injections of Capan-1 or HPAF-II pancreatic cancer cells. [file 13046_2020_1822_MOESM1_ESM.pptx]

## Slide 1
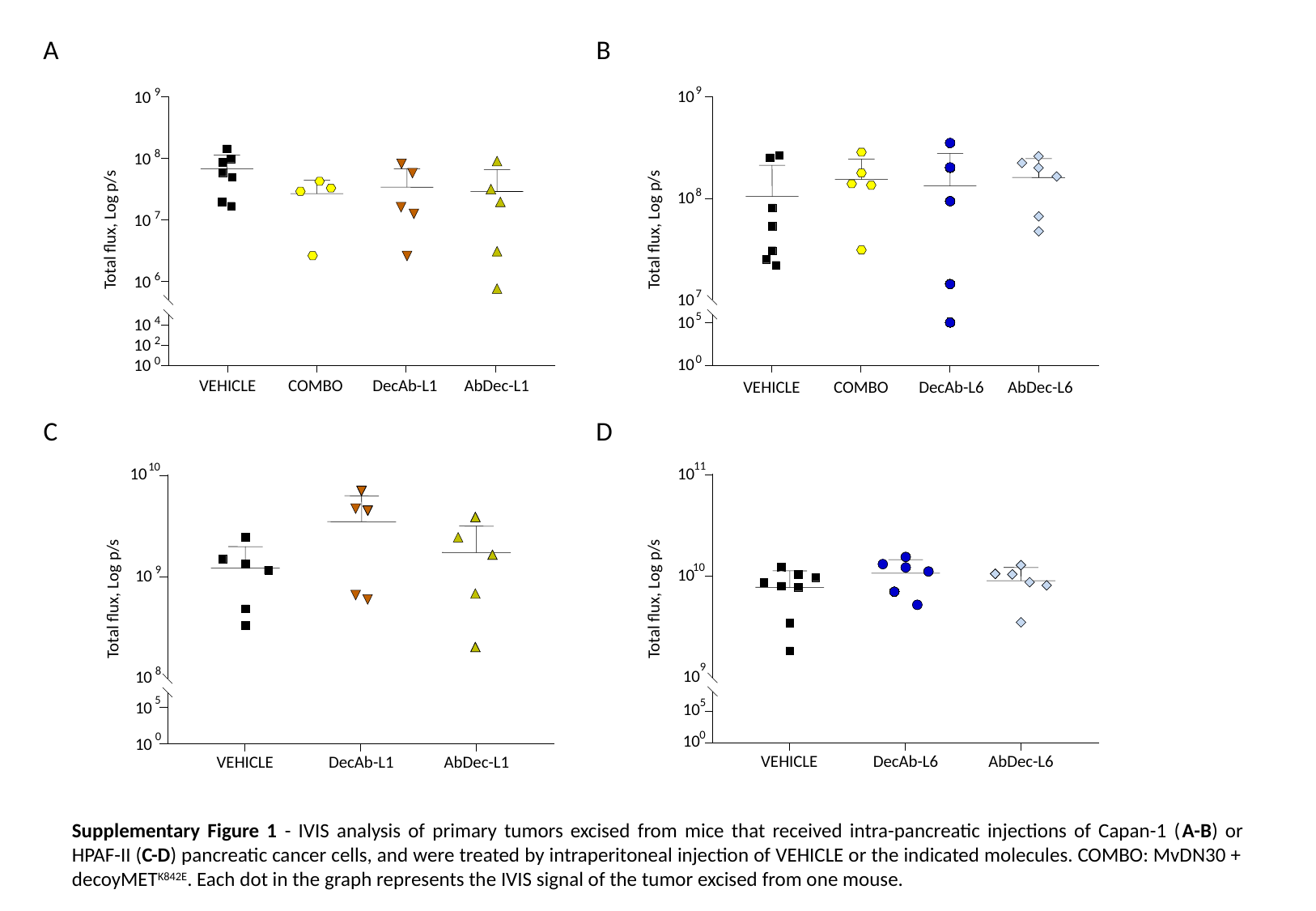

A
9
10
8
10
Total flux, Log p/s
7
10
6
10
4
10
2
10
0
10
VEHICLE
COMBO
DecAb-L1
AbDec-L1
B
9
10
Total flux, Log p/s
8
10
7
10
5
10
0
10
VEHICLE
COMBO
DecAb-L6
AbDec-L6
C
D
11
10
10
Total flux, Log p/s
9
10
8
10
5
10
0
10
VEHICLE
DecAb-L1
AbDec-L1
10
Total flux, Log p/s
10
10
9
10
5
10
0
10
VEHICLE
DecAb-L6
AbDec-L6
Supplementary Figure 1 - IVIS analysis of primary tumors excised from mice that received intra-pancreatic injections of Capan-1 (A-B) or HPAF-II (C-D) pancreatic cancer cells, and were treated by intraperitoneal injection of VEHICLE or the indicated molecules. COMBO: MvDN30 + decoyMETK842E. Each dot in the graph represents the IVIS signal of the tumor excised from one mouse.
