# Supplementary material for: A receptor-antibody hybrid hampering MET-driven metastatic spread
Source: J Exp Clin Cancer Res. 2021 Jan 14;40:32. doi: 10.1186/s13046-020-01822-5 (PMC7807714; doi:10.1186/s13046-020-01822-5)
Supplement: Supplementary file 2 — Additional file 2: Supplementary Fig. 2. IVIS images of livers excised from hHGF-ki mice that received intra-pancreatic injection of Capan-1 cells. [file 13046_2020_1822_MOESM2_ESM.pptx]

## Slide 1
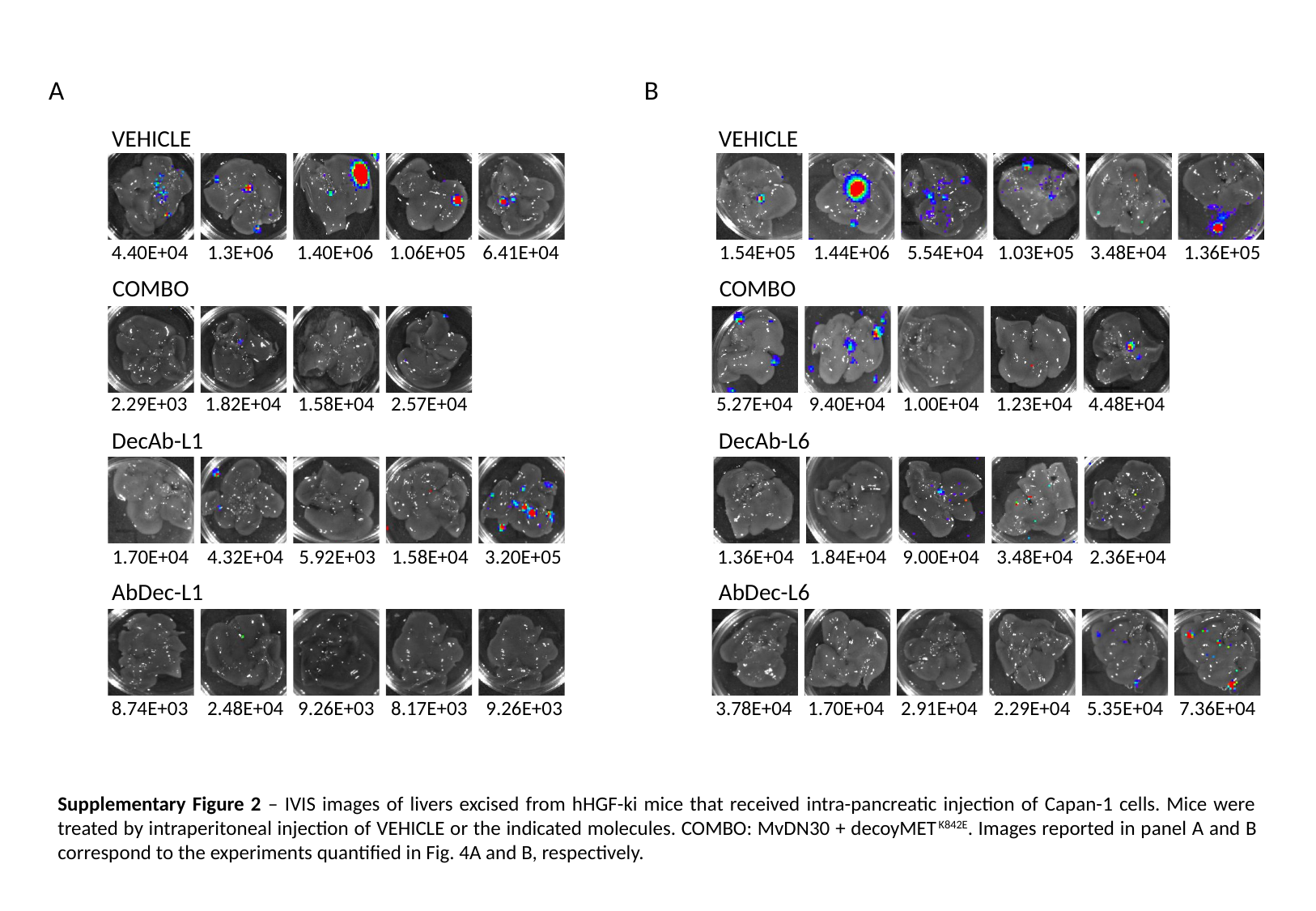

A
VEHICLE
4.40E+04
1.3E+06
1.40E+06
1.06E+05
6.41E+04
COMBO
2.29E+03
1.82E+04
1.58E+04
2.57E+04
DecAb-L1
1.70E+04
4.32E+04
5.92E+03
1.58E+04
3.20E+05
AbDec-L1
8.74E+03
2.48E+04
9.26E+03
8.17E+03
9.26E+03
B
VEHICLE
1.54E+05
1.44E+06
5.54E+04
1.03E+05
3.48E+04
1.36E+05
COMBO
5.27E+04
9.40E+04
1.00E+04
1.23E+04
4.48E+04
DecAb-L6
1.36E+04
1.84E+04
9.00E+04
3.48E+04
2.36E+04
AbDec-L6
3.78E+04
1.70E+04
2.91E+04
2.29E+04
5.35E+04
7.36E+04
Supplementary Figure 2 – IVIS images of livers excised from hHGF-ki mice that received intra-pancreatic injection of Capan-1 cells. Mice were treated by intraperitoneal injection of VEHICLE or the indicated molecules. COMBO: MvDN30 + decoyMETK842E. Images reported in panel A and B correspond to the experiments quantified in Fig. 4A and B, respectively.
