# Supplementary material for: A receptor-antibody hybrid hampering MET-driven metastatic spread
Source: J Exp Clin Cancer Res. 2021 Jan 14;40:32. doi: 10.1186/s13046-020-01822-5 (PMC7807714; doi:10.1186/s13046-020-01822-5)
Supplement: Supplementary file 3 — Additional file 3: Supplementary Fig. 3. IVIS images of lungs excised from hHGF-ki mice that received intra-pancreatic injection of HPAF-II cells. [file 13046_2020_1822_MOESM3_ESM.pptx]

## Slide 1
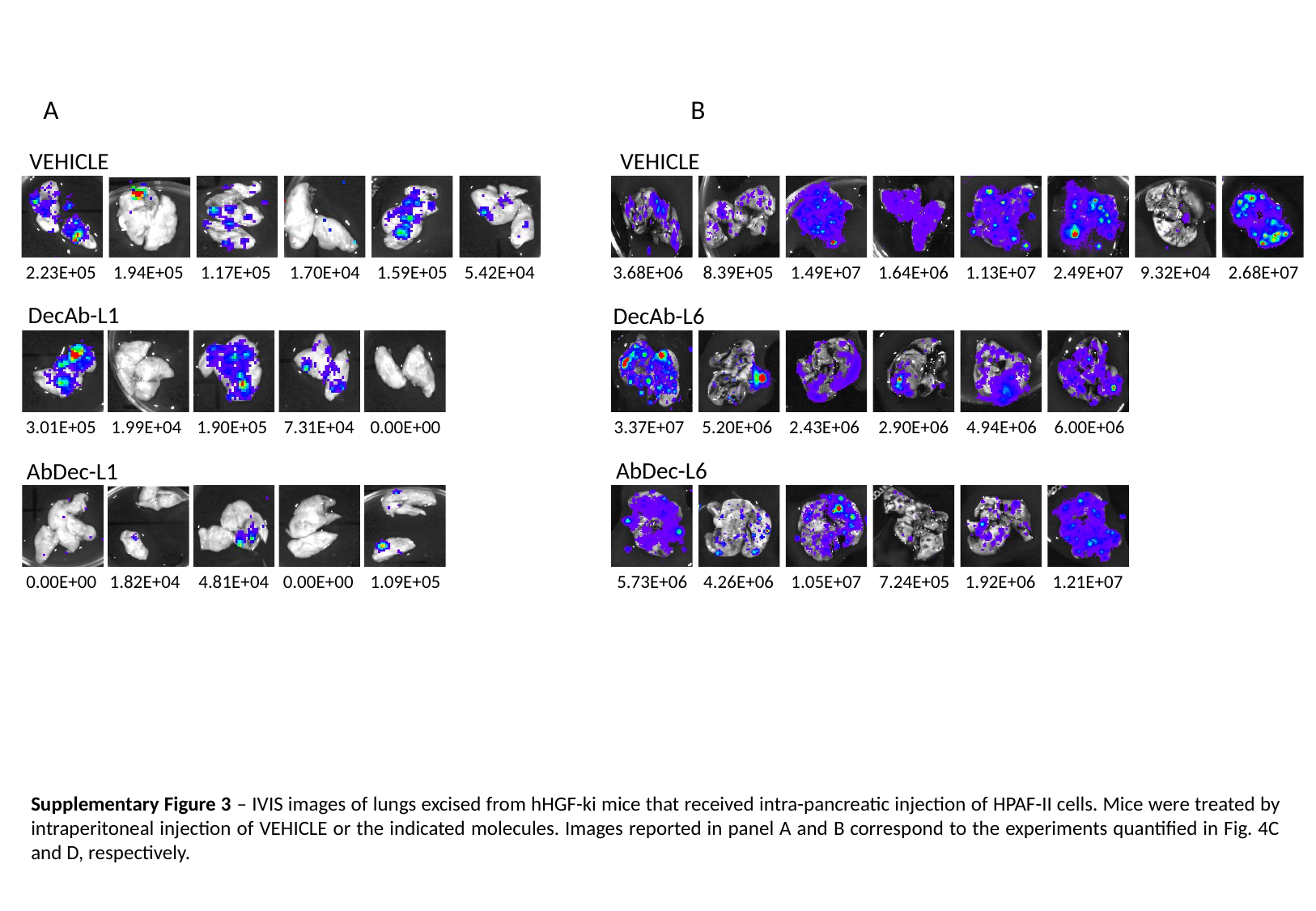

A
B
VEHICLE
VEHICLE
2.23E+05
1.94E+05
1.17E+05
1.70E+04
1.59E+05
5.42E+04
3.68E+06
8.39E+05
1.49E+07
1.64E+06
1.13E+07
2.49E+07
9.32E+04
2.68E+07
DecAb-L1
DecAb-L6
3.01E+05
1.99E+04
1.90E+05
7.31E+04
0.00E+00
3.37E+07
5.20E+06
2.43E+06
2.90E+06
4.94E+06
6.00E+06
AbDec-L6
AbDec-L1
0.00E+00
1.82E+04
4.81E+04
0.00E+00
1.09E+05
5.73E+06
4.26E+06
1.05E+07
7.24E+05
1.92E+06
1.21E+07
Supplementary Figure 3 – IVIS images of lungs excised from hHGF-ki mice that received intra-pancreatic injection of HPAF-II cells. Mice were treated by intraperitoneal injection of VEHICLE or the indicated molecules. Images reported in panel A and B correspond to the experiments quantified in Fig. 4C and D, respectively.
