# Supplementary material for: A receptor-antibody hybrid hampering MET-driven metastatic spread
Source: J Exp Clin Cancer Res. 2021 Jan 14;40:32. doi: 10.1186/s13046-020-01822-5 (PMC7807714; doi:10.1186/s13046-020-01822-5)
Supplement: Supplementary file 4 — Additional file 4: Supplementary Fig. 4. Immunohistochemical analysis of MET phosphorylation in pancreatic primary tumors treated with AbDec-L1. [file 13046_2020_1822_MOESM4_ESM.pptx]

## Slide 1
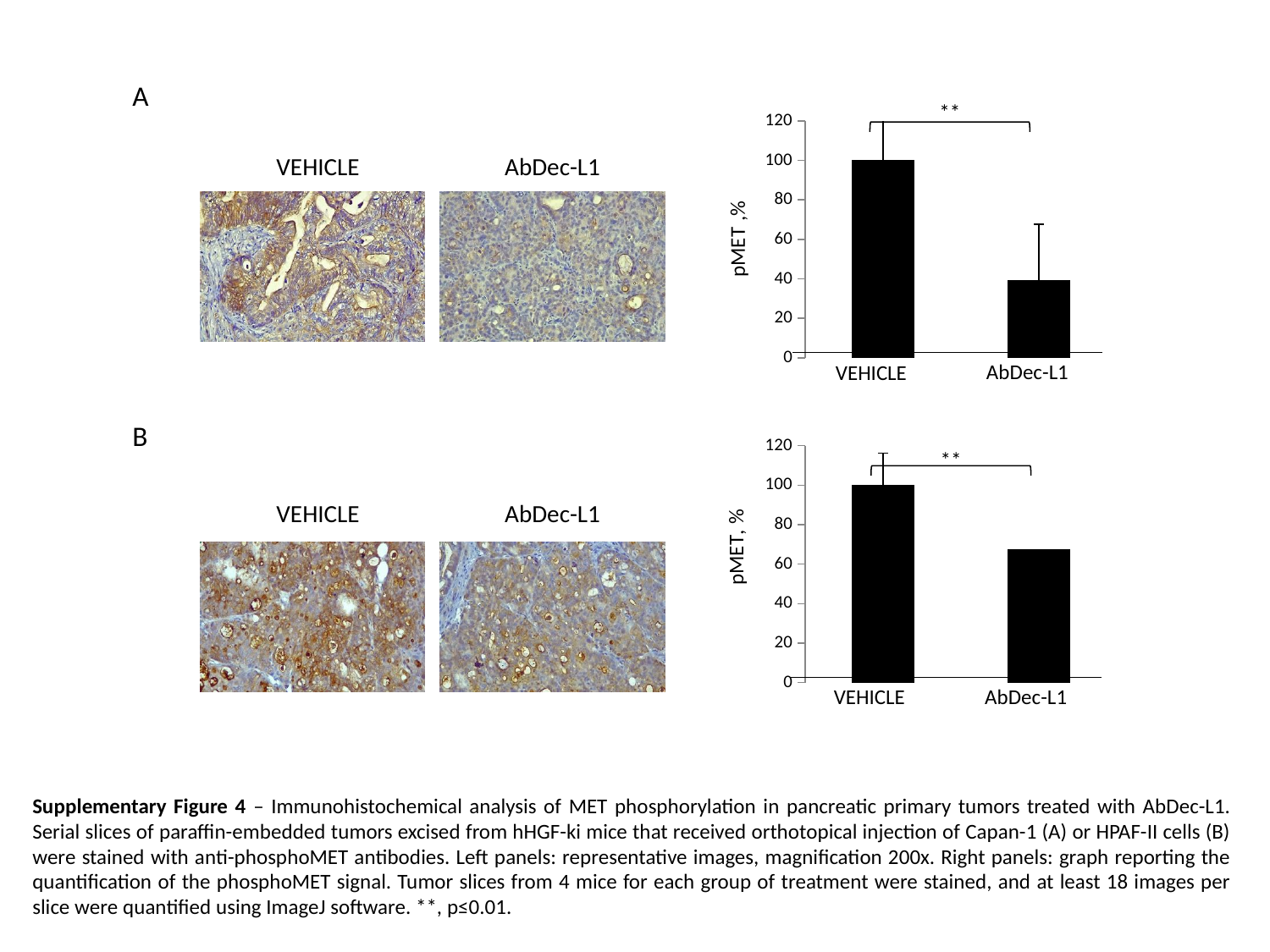

A
**
### Chart
| Category | |
|---|---|
| Vehicle | 100.0 |
| L3 M-K | 39.4629852478036 |VEHICLE
AbDec-L1
AbDec-L1
VEHICLE
B
### Chart
| Category | |
|---|---|
| Vehicle | 100.0 |
| L3 M-K | 67.7320585854876 |
**
VEHICLE
AbDec-L1
10X
AbDec-L1
VEHICLE
Supplementary Figure 4 – Immunohistochemical analysis of MET phosphorylation in pancreatic primary tumors treated with AbDec-L1. Serial slices of paraffin-embedded tumors excised from hHGF-ki mice that received orthotopical injection of Capan-1 (A) or HPAF-II cells (B) were stained with anti-phosphoMET antibodies. Left panels: representative images, magnification 200x. Right panels: graph reporting the quantification of the phosphoMET signal. Tumor slices from 4 mice for each group of treatment were stained, and at least 18 images per slice were quantified using ImageJ software. **, p≤0.01.
