# Supplementary material for: A receptor-antibody hybrid hampering MET-driven metastatic spread
Source: J Exp Clin Cancer Res. 2021 Jan 14;40:32. doi: 10.1186/s13046-020-01822-5 (PMC7807714; doi:10.1186/s13046-020-01822-5)
Supplement: Supplementary file 5 — Additional file 5: Supplementary Fig. 5. IVIS images of organs excised from hHGF-ki mice that received sub-cuteaneous injection of CL-901 cells. [file 13046_2020_1822_MOESM5_ESM.pptx]

## Slide 1
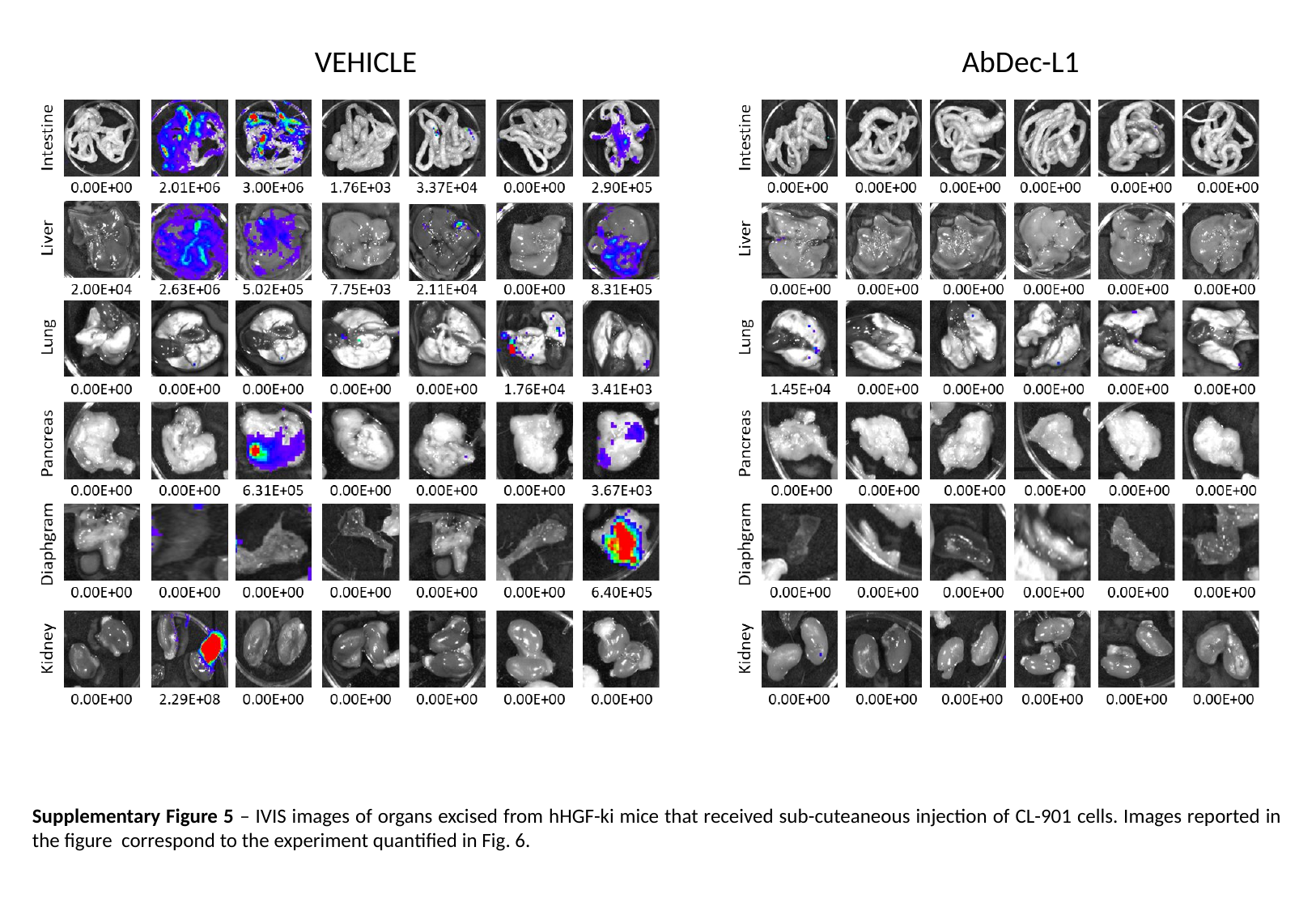

VEHICLE
AbDec-L1
Supplementary Figure 5 – IVIS images of organs excised from hHGF-ki mice that received sub-cuteaneous injection of CL-901 cells. Images reported in the figure correspond to the experiment quantified in Fig. 6.
