# Supplementary material for: A receptor-antibody hybrid hampering MET-driven metastatic spread
Source: J Exp Clin Cancer Res. 2021 Jan 14;40:32. doi: 10.1186/s13046-020-01822-5 (PMC7807714; doi:10.1186/s13046-020-01822-5)
Supplement: Supplementary file 6 — Additional file 6: Supplementary Fig. 6. Analysis of CL-901 primary tumors treated with AbDec-L1. [file 13046_2020_1822_MOESM6_ESM.pptx]

## Slide 1
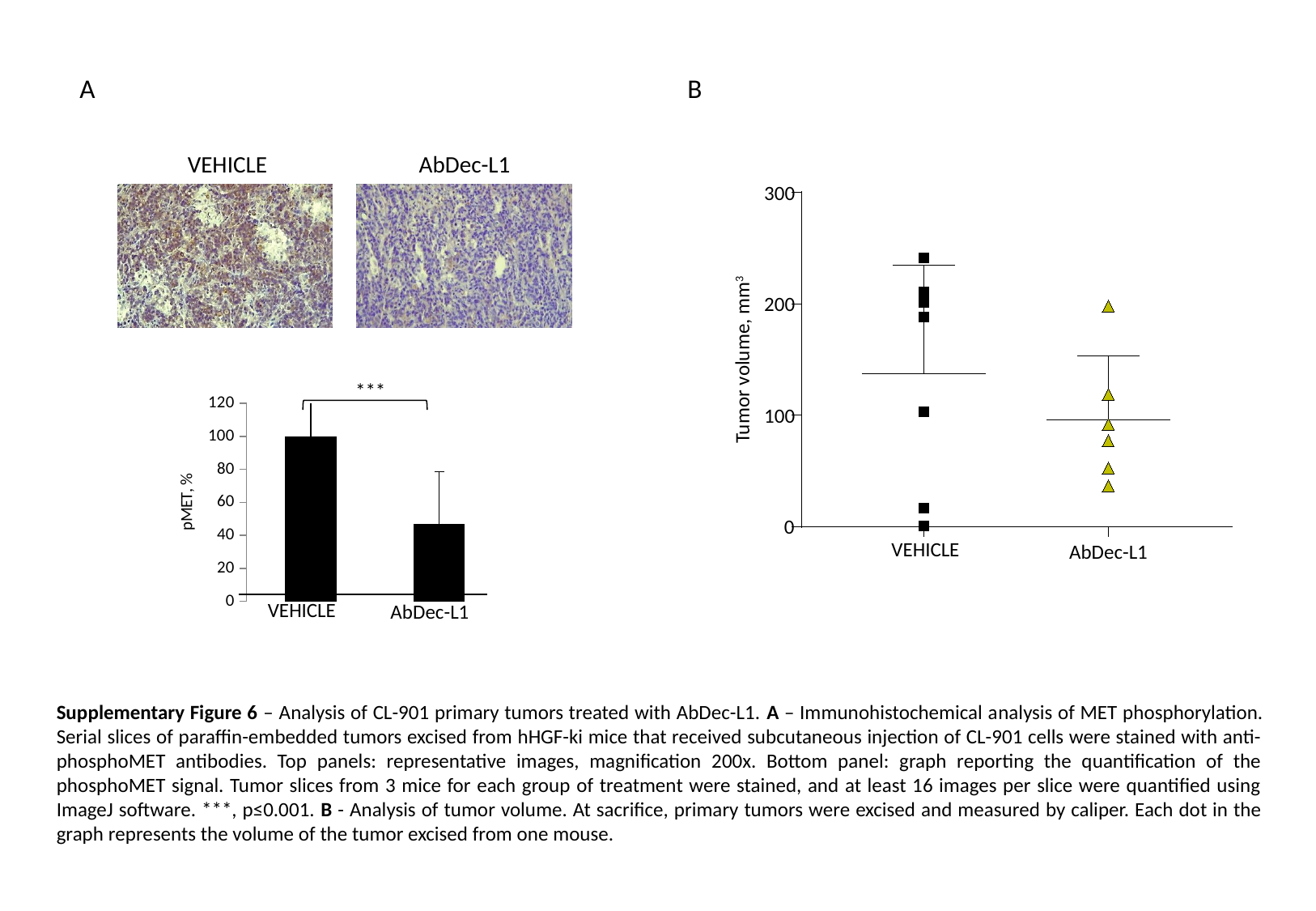

A
B
VEHICLE
AbDec-L1
***
### Chart
| Category | |
|---|---|
| Vehicle | 100.0 |
| L3 M-K | 46.97096371423772 |VEHICLE
AbDec-L1
300
200
Tumor volume, mm3
100
0
VEHICLE
AbDec-L1
Supplementary Figure 6 – Analysis of CL-901 primary tumors treated with AbDec-L1. A – Immunohistochemical analysis of MET phosphorylation. Serial slices of paraffin-embedded tumors excised from hHGF-ki mice that received subcutaneous injection of CL-901 cells were stained with anti-phosphoMET antibodies. Top panels: representative images, magnification 200x. Bottom panel: graph reporting the quantification of the phosphoMET signal. Tumor slices from 3 mice for each group of treatment were stained, and at least 16 images per slice were quantified using ImageJ software. ***, p≤0.001. B - Analysis of tumor volume. At sacrifice, primary tumors were excised and measured by caliper. Each dot in the graph represents the volume of the tumor excised from one mouse.
