# Supplementary material for: A receptor-antibody hybrid hampering MET-driven metastatic spread
Source: J Exp Clin Cancer Res. 2021 Jan 14;40:32. doi: 10.1186/s13046-020-01822-5 (PMC7807714; doi:10.1186/s13046-020-01822-5)
Supplement: Supplementary file 7 — Additional file 7: Supplementary Fig. 7. Serum concentration of AbDec-L1 after single i.v. administration to Sprague Dawley rats at different time points. [file 13046_2020_1822_MOESM7_ESM.pptx]

## Slide 1
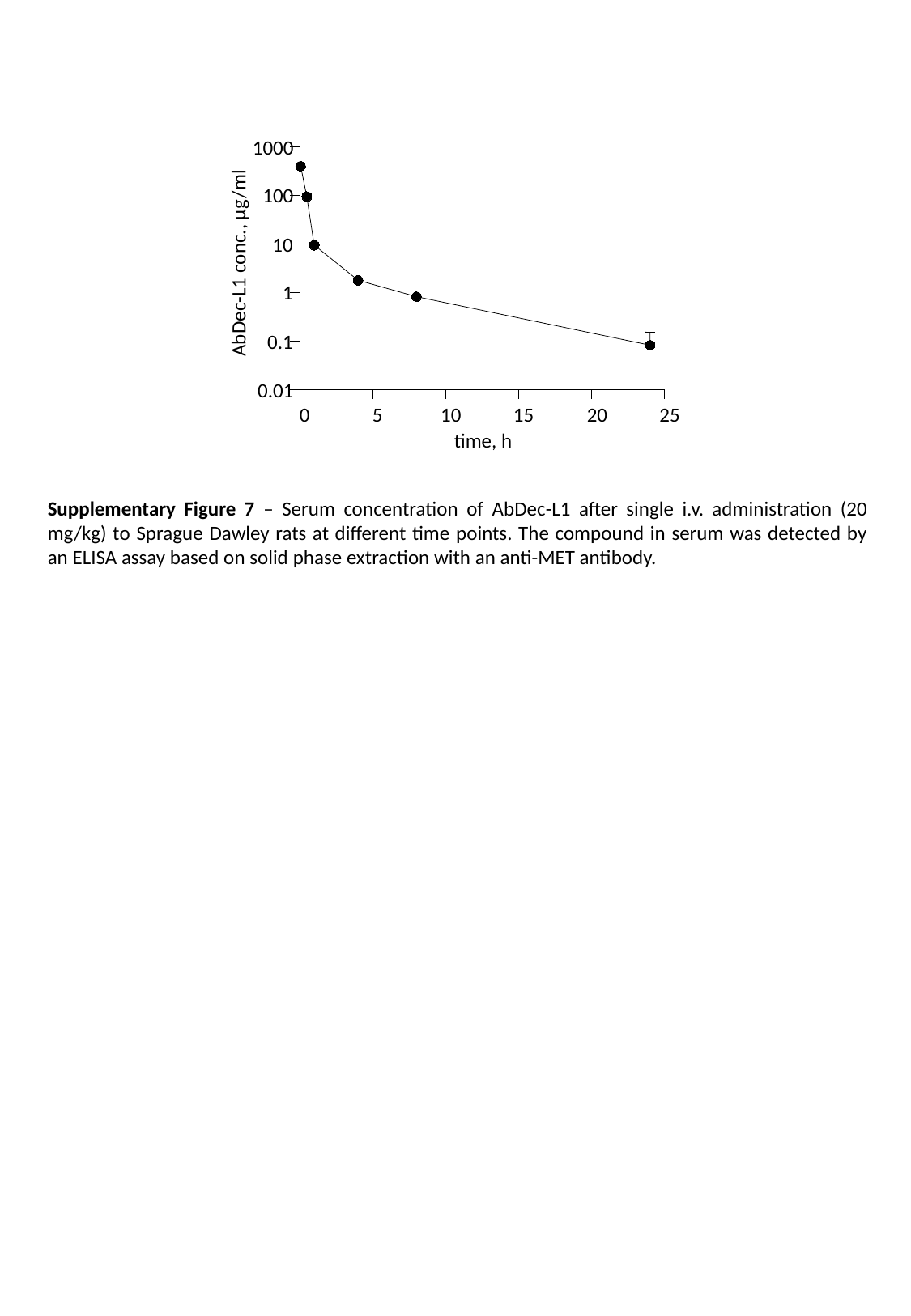

1000
100
10
AbDec-L1 conc., µg/ml
1
0.1
0.01
0
5
10
15
20
25
time, h
Supplementary Figure 7 – Serum concentration of AbDec-L1 after single i.v. administration (20 mg/kg) to Sprague Dawley rats at different time points. The compound in serum was detected by an ELISA assay based on solid phase extraction with an anti-MET antibody.
